# Primate Drum Kit: A System for Studying Acoustic Pattern Production by Non-Human Primates Using Acceleration and Strain Sensors

**DOI:** 10.3390/s130809790

**Published:** 2013-07-31

**Authors:** Andrea Ravignani, Vicente Matellán Olivera, Bruno Gingras, Riccardo Hofer, Carlos Rodríguez Hernández, Ruth-Sophie Sonnweber, W. Tecumseh Fitch

**Affiliations:** 1 Department of Cognitive Biology, University of Vienna, Althanstrasse, 14, Vienna A-1090, Austria; E-Mails: bruno.gingras@univie.ac.at (B.G.); riccardo.hofer@univie.ac.at (R.H.); ruth-sophie.sonnweber@univie.ac.at (R.S.); tecumseh.fitch@univie.ac.at (T.F.); 2 Escuela de Ingeniería Industrial e Informática, Universidad de León, 24071 León, Spain; E-Mails: vicente.matellan@unileon.es (V.M.); carlosrhrh@hotmail.com (C.R.)

**Keywords:** accelerometer, piezoelectric sensor, chimpanzee, drumming, evolution of music, cognition, primate, sonification, animal-computer interaction, human-computer interaction

## Abstract

The possibility of achieving experimentally controlled, non-vocal acoustic production in non-human primates is a key step to enable the testing of a number of hypotheses on primate behavior and cognition. However, no device or solution is currently available, with the use of sensors in non-human animals being almost exclusively devoted to applications in food industry and animal surveillance. Specifically, no device exists which simultaneously allows: (i) spontaneous production of sound or music by non-human animals via object manipulation, (ii) systematical recording of data sensed from these movements, (iii) the possibility to alter the acoustic feedback properties of the object using remote control. We present two prototypes we developed for application with chimpanzees (*Pan troglodytes*) which, while fulfilling the aforementioned requirements, allow to arbitrarily associate sounds to physical object movements. The prototypes differ in sensing technology, costs, intended use and construction requirements. One prototype uses four piezoelectric elements embedded between layers of Plexiglas and foam. Strain data is sent to a computer running Python through an Arduino board. A second prototype consists in a modified Wii Remote contained in a gum toy. Acceleration data is sent via Bluetooth to a computer running Max/MSP. We successfully pilot tested the first device with a group of chimpanzees. We foresee using these devices for a range of cognitive experiments.

## 1. Introduction

### 1.1. The Need for Sensing Technologies in Cognitive Research

Humans, from their birth onwards, are characterized by a strong tendency to explore the space surrounding them [1]. An important part of this exploratory behavior consists in the feedback obtained, generally of acoustic and tactile nature [1,2]. Recently, increase in computational power and low cost sensing devices encouraged the construction of projects linking movement and sound: *movement sensing* can trigger *auditory feedback* in several possible ways [3–6]. Especially in the fields of human cognitive science and artistic performance, scientists and artists have pioneered an array of approaches differing in sensing technologies and purposes [5,7,8]. The accelerometer contained in the Nintendo Wii Remote or Microsoft Kinect's infrared sensors, for instance, can provide a cheap, consumer-level sensing option [6–10]. Freely downloadable libraries are constantly developed to interface these sensors with computers [9]. Finally, visual programming languages like Max/MSP are an accessible possibility for project developers with little or no programming experience [4,5,11].

In parallel, sensing in the animal kingdom has exclusively focused on applications related to food industry and measuring animal behavior [12–14]. The emerging field of animal cognition, however, requires more advanced sensing possibilities than what is currently implemented. An increasing number of laboratories are training non-human animals to the use of touch screens in order to easily collect the animals' responses [15–18]. However, cognitive scientists working with animals are becoming more and more interested in the *production abilities* of their study species. Inspired by the cognitive gap between perception and production in humans, researchers wonder whether a similar divide can be found in other animal species. Non-human primates are among the species most likely to equal many human cognitive abilities. Chimpanzees, our closest living relatives, have already shown human-like skills in several cognitive domains [17,19]. Among chimpanzees, high-ranking males are known for their dominance displays, often consisting in striking objects in order to generate loud, prominent sounds [20]. Similarly, chimpanzees' playful behavior may involve sound production.

Chimpanzees have been reported to perceive some features of music [21], though whether they would be able to actively produce musical patterns is a topic of debate and conjectures [22]. Most evidence on rhythmic production in chimpanzees is based on observational studies and anecdotes, and no well-controlled data recording has been accomplished due, among others, to the absence of adequate technological resources [20,23]. Similarly, multimodal entrainment to a musical beat relies on an auditory-motor brain circuit and is thought to be only present in humans, a few mammalian species and some bird species [24]. Rigorously testing the presence of rhythmic abilities in chimpanzees will be fundamental to understand what is unique in human cognition [23,25]. This, in turn, can only be achieved with adequate sensing and computational tools.

### 1.2. State of the Art

Our prototypes are the first attempt at bridging two strands of research that have been, to our knowledge, completely disconnected until now. On the one hand, electronic sensory devices are used in humans for scientific and expressive purposes, be it in cognitive research [7], data analysis [26] or music performance [27]. On the other hand, sensors are broadly used in non-human animals, though almost exclusively to monitor behavioral patterns and welfare [12–14].

Over the last decades, an impressive amount of research has been devoted to the sonification of data sets or sensory readings collected in real time [26]. Mapping sensory readings to sounds can have a number of applications, such as accurate tracking of vital functions during surgery [28], augmented human interaction with objects [29], and facilitation in learning new movements [2,4]. In particular, sensing for sound manipulation purposes has proven critical in developing new musical interfaces and means of expression [5,27,30,31]. Sensory technologies and computational power have enabled the development of virtual musical instruments and augmented reality environments [6,10,11,32]. Unfortunately, almost all auditory interaction technologies developed until now have focused on humans as potential users. In some cases, one could use human-specific sensors for animal purposes [19]. However, usability and species-appropriateness of the technologies used should be taken into account [33–36]: inappropriate interfaces could just be unused by, or not endure the strength of, the particular animal species.

Most technologies and sensory networks developed until now for animals have been concerned with appropriateness and species-specificity of the devices. One main area of this research deals with monitoring behavior of groups of animals [37–41]. Sensor networks are also used to monitor health and welfare in non-human animals using readings of body temperature and other physiological variables [42–47]. Quite recently, however, researchers in human-computer interaction have started to discuss the importance of developing paradigms for (non-human) animal-computer interaction [34–36]. Specific interfaces have been built so that cows [48], hamsters [49] and dogs [50,51] could interact with a computer, either aurally or visually. Apart from these few cases, the field of animal-computer interaction is just beginning to develop, and promises contributions to sustainability, cognition and adaptability of technologies to specific needs of some human groups, such as pre-linguistic infants or elderly adults [36]. In particular, the field of animal cognition has proved its strong interest in developing technological testing paradigms adapted to particular species and tasks [15,16,18,52,53].

### 1.3. Filling a Technological Gap

This paper describes two innovative prototypes we specifically developed for sensing movements in chimpanzees and mapping them to specific sounds in real time [23]. To our knowledge, this is the first attempt at building this type of technological tool specifically designed for primate research.

The devices we present here fill the aforementioned technological gap in animal cognition by taking advantage of chimpanzees' spontaneous behavior. We developed two prototypes whose manipulation by chimpanzees produces sounds, which can be altered depending on the specific experiment taking place. Our prototypes rely on two main principles: *movement sensing* and *sonification*. Acceleration and strain sensors are embedded into manipulable objects. A computer receives the measurements, processes the data and sonifies it. According to Kramer *et al.*, “Sonification is defined as the use of nonspeech audio to convey information. More specifically, sonification is the transformation of data relations into perceived relations in an acoustic signal for the purposes of facilitating communication or interpretation” [54]. Combining the sensing with the sonification principle enables a real time mapping between movements and sounds. Therefore, one can alter the physical-acoustic characteristics of an object by assigning particular, experimentally manipulated sounds to its displacement.

This paper is organized as follows. We explain the need of animal sonification devices for cognitive research. We give an overview of the current technologies available and mention a number of desiderata such devices should have. We illustrate the overall design features of our prototypes and then describe each of them in detail.

## 2. Description of the Problem

### 2.1. Overview

**Figure 1.**
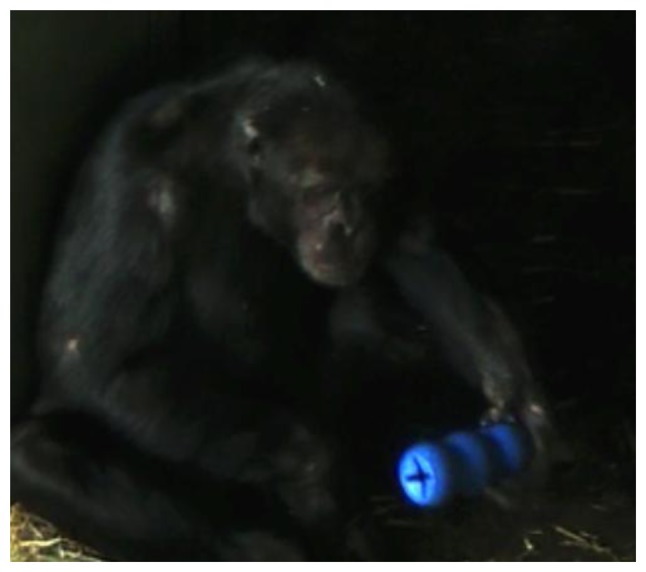
Chimpanzee manipulating a Kong Toy, constituting the outer shell of one of our prototypes.

The use of sensors in non-human animals is almost exclusively devoted to livestock and animal surveillance applications. The constantly growing field of animal cognition, however, requires ever finer and more specific sensing possibilities. In particular, a number of important hypotheses in primate cognition deal with the connection between movement and auditory perception [22,23,25]. In order to show one of these, rhythmic production abilities in chimpanzees, we developed two prototype devices specifically adapted to this species (Figure 1) [23,55].

Each prototype presented here is composed of a sensing and a feedback unit, and allows to arbitrarily associate sounds to physical movements. The sensing unit sends acceleration or strain data to a computer, which converts them into sound and plays it in real time. Our prototypes, apart from constituting a first attempt at animal-computer cognitive interaction, will allow rigorous testing of a number of hypotheses in primate cognition [22,23,25]. Moreover, we plan to make the software freely available, so that interested researchers can benefit from our findings.

### 2.2. Desiderata and Constraints

The devices we developed satisfy a number of desiderata, plus a crucial tradeoff between ergonomics relative to chimpanzee behavior, and efficiency of the device when used to conduct scientific experiments (see Table 1).

More specifically, desiderata and constraints imposed on the construction of the device affect both the sensing and the computation components of the project. The sensing part has to be resistant, modular, low-voltage, weather resistant, hence making it suitable for safe primate work in general. Moreover, it should be interesting for the primate, possibly inexpensive and easy to connect and configure. The software part requires quick data processing; therefore the computations performed must be moderate in number and nature, so as to limit the computational load.

### 2.3. Requirements: Sensing

Chimpanzees are capable of exerting strong force by hitting and tossing objects. Their muscular strength has been reported to be several times that of a human being [56]. Chimpanzees are supposed to have free access to the device and expected to vigorously manipulate it. Hence, the sensing part of the device has to be resistant to strain and extreme shocks while remaining sensitive to the physical variations of interest. It may be that the animal succeeds nonetheless in breaking the device. Bearing in mind this possibility, the voltage, which the animal may come in contact with, should be kept to a bare minimum. This would avoid eventual electrocution of the animal and short-circuiting the electronics in case of moist environment.

In the eventuality of damage or strong pulling, the device needs to be modular. If a part of the device is damaged or removed, it is desirable that only the damaged component be replaced. Similarly, if the pulling force exerted on the sensor is strong enough for the entire device to be carried away, only a module should detach, leaving the main part of the device undamaged. Considering all these eventualities, the modules composing the device need to be inexpensive and easy to find and replace. Damage and destruction of parts are concrete possibilities.

Chimpanzees are usually housed with access to outdoor enclosures. The device has to withstand, in principle, any kind of meteorological condition. This means resistance both to long periods of direct sunlight and heat, and to rain and cold weather. Other desiderata concern the ease of preparing the device before any measure can be taken or an experiment run. The scientist should be able to easily and quickly activate the device, connect it to a computer and start recording data.

Finally, in order to obtain any data at all, the device has to incite the primate to manipulation and play. Hence its physical properties need to be such that, before receiving any auditory feedback provoked by a manipulation, the chimpanzees will be spurred to approach the device and play with it.

### 2.4. Requirements: Processing

The software should handle a possibly continuous stream of data. Critically, the processing part of the device needs to concurrently handle the storage, processing and playback of the very same signal. Moreover, the mapping of physical variations in the sensor to sounds must be flexible and easily adjustable. There are types of sounds that chimpanzees are most interested in and which can be used to induce interest in manipulating the object [21,57]. Similarly, testing specific hypotheses requires particular sounds and inter-onset intervals. Therefore the researcher needs to be able to adjust the acoustic properties of the feedback as fast as possible. Finally, object manipulation and auditory feedback should be perceived as causally related. Therefore, all computations must occur as close as possible to real time.

### 2.5. Requirements: Usability and Experience

The device should also satisfy a number of requirements from the chimpanzee's perspective as a user. From the point of view of *usability*, the movement to sound mapping should be relatively straightforward, spurring the animal to interact. Hence, typical movements chimpanzees may perform must be accounted for, such as the strong hits during pant-hoot displays or finger poking while exploring objects. The amount of learning needed to use the device should be minimal. This will likely be granted by chimpanzees' neophilia and exploratory attitude [58].

Regarding the chimpanzees' *experience*, the device should be physically reactive and acoustically satisfactory. If chimpanzees push the device, it should bounce back, hence spurring the animals to push it once again. When chimpanzees are displaying and hitting on it, it should respond with loud sounds.

Crucially, using the device should grant a sense of satisfaction and reward by itself, to ensure that the chimpanzees will return to it over time. In a number of species vocal communication correlates with the regulation of the dopaminergic system, which is in turn connected with reward and satisfaction [59,60]. In different bird species the perception and production of songs are tightly linked with reward systems [61]. Similarly, when humans listen to music reward pathways are active [62]. To our knowledge, no research has been done on sound preference in chimpanzees. Hence, the prototype should be able to play a range of sounds, so that it is possible to heuristically adjust the movement-sound mapping to chimpanzees' preferences. This way, sonic interaction will be rewarding in itself and possibly serve as an enrichment for captive animals.

### 2.6. Experimental Requirements

From the point of view of the experimenter, two requirements seem particularly important. The software should be flexible and expandable, so that parameters can be changed and new modules and functionalities added, depending on the particular use envisaged. Moreover, the entire apparatus should be easy to use, so that even researchers with limited technical experience can run an experimental session.

## 3. General Design Features

**Figure 2.**
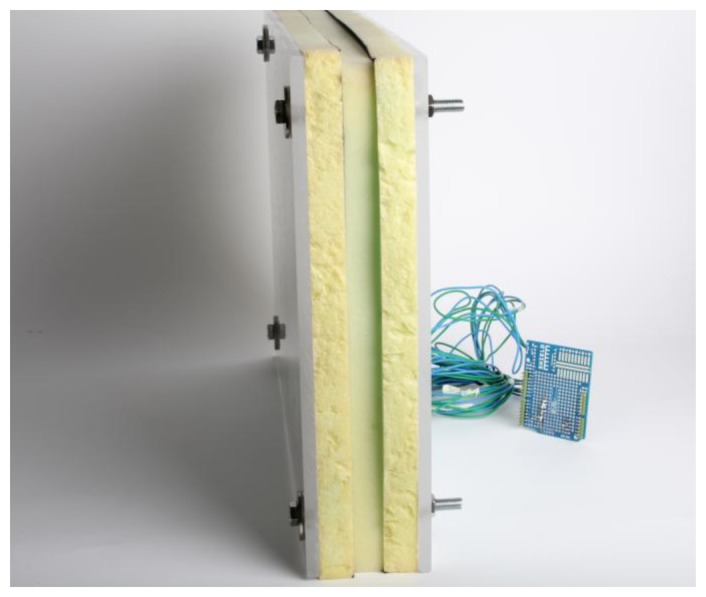
The wired prototype. The rightmost part can be mounted on a chimpanzee enclosure. The animals come in contact with the leftmost side.

The two prototype devices we developed differ in size, connectivity and sensing technology. Our *wired prototype* consists in piezoelectric sensors embedded in a parallelepiped, connected to a computer via an Arduino board (Figure 2). Our *wireless device* is made of a hollow gum toy containing a videogame controller (Wii Remote [9], www.nintendo.com), which is connected to a Mac computer via Bluetooth (Figure 6). Each prototype is described in detail in the next two sections. Here we outline their general, common features.

### 3.1. Data Collection

The general concept consists in embedding a sensor in a manipulable object. The object is designed to incite chimpanzees to interaction and play [63]. The physical characteristics and design of the sensory part are dictated by scrutinizing videos of chimpanzees playing with objects, so as to maximize the chances of interaction with our prototypes [55]. The sensors in the object record physical variations (strain or motion). Depending on the prototype, sensory data can go through a phase of preprocessing before reaching the computer. Preprocessing is done using an Arduino board (http://www.arduino.cc/), which, apart from the analog/digital conversion, is in charge of basic filtering. Once the data reaches the computer, processing is accomplished using different pieces of software. Depending on the sensor and prototype, data is elaborated using patches written in Max/MSP (http://cycling74.com/products/max/) or scripts written in the multi-platform, freely available programming language Python (www.python.org).

### 3.2. Data Processing

The software processing part is responsible for four key tasks: filtering the data, extracting parameters relevant to sound production, logging specific occurrences or variations of the parameters, and playing sounds corresponding to critical parameters' states (as specified by the user).

Our basic *data filtering* method simply discards sensory readings below a specified threshold. This simple system allows nonetheless for a rough, though efficient, data classification for a low computational cost. Parameters of interest are successively extracted through concurrent computation of thresholds on sensor input and time.

The *time thresholding* allows for refractory periods in outputting the parameter. When an activation threshold is crossed and the system produces a sound, the system waits for a specified number of milliseconds before resuming its activity. This entails that, once a sound has been produced by the system, sensory readings can trigger new sounds only after a given time period. Such design choice makes it possible that only one sound gets played each time the object is manipulated, therefore avoiding misleading repetitions.

*Logging* variations in the parameters of interest is essential. No matter if the interactions with the device are observed or videotaped, a precise log containing the exact time and magnitude of the interaction is crucial in any scientific study. Therefore, both prototypes feature the possibility of saving data to a text file in CSV (comma separated value) format. This can be later read in Microsoft Excel or parsed by any programming language in order to extract meaningful statistics. Moreover, if the interactions are videotaped, information in the log can be later integrated with the video.

Finally, *real-time playback* is one of the key features of our prototypes. Our software is extremely flexible, when it comes both to configuration and expansion. Thresholds on parameters and settings can be altered to vary the sensitivity of the device and the properties of the sounds played. Additionally, the mapping between raw data, parameters and sound output can be changed at will by modifying few lines of code or Max objects. Once the computer has produced the sound, the signal is transmitted to an external loudspeaker and one manipulation-auditory feedback interaction is completed.

## 4. Wired Prototype

This prototype was designed to sonify a chimpanzee's common behavior, namely hitting on walls or objects using either the upper or lower limbs. This prototype can be hence used as a “sonically augmented” substitute for the usual resonant surface [29]. The ricochet property of this device (see *Sensory Module*) satisfies chimpanzees' natural behavioral predispositions to hit and push objects in order to generate sound [20]. Additionally, its acoustic feedback properties can be artificially manipulated, so to sound louder than the usual surfaces chimpanzees usually hit.

The Wired device is composed of a sensory module, a custom produced board, an Arduino board and a computer with a USB interface. This device is supposed to be mounted vertically, resting towards a wall or bars of chimpanzees' enclosures.

This prototype has a number of valuable features. Most prominently, it is built with inexpensive and relatively easy to find components. Assembling the device requires only moderate craftsmanship and electronic skills and the use of a few tools (drill, saw, *etc.*). This prototype is extremely safe concerning possibilities of electrocution. No electricity is required to operate its sensing module: the only electricity present is the one produced by the piezoelectric sensors under strain and therefore has negligible voltage.

While building this device and programming its software, we deemed important for it to be able to discriminate between types of mechanical stress. There are three main ways in which an individual can interact with this prototype: by simply touching it, by tapping on it, and finally by pushing it (until provoking the displacement of the upper Plexiglas layer). We decided that pushing was the only type of strain to which sounds would be associated. This discrimination was achieved both by selecting piezoelectric sensors with specific electrical characteristics and real-time data filtering with our software (see Sections 4.1 and 4.5).

### 4.1. Wired Sensory Module

The sensory module is a parallelepiped with dimensions 420 mm × 300 mm × 76 mm (volume: 9.576 L). The parallelepiped is made of two layers of Plexiglas (dimensions: 420 mm × 300 mm × 8 mm each) encasing three layers of solid foam (dimensions: 420 mm × 300 mm × 20 mm each). 40 mm away from each corner, four screws are passed through the Plexiglas and foam, and fixed with a die at one of their ends. Longer screws enable the parallelepiped to be fixed to a wall or any vertical surface required for acquiring data. The screws serve the function of holding the module together and, combined with the foam, confer it a ricochet property once the Plexiglas is pushed. It is essential for the module to rebound once pressed, so that the chimpanzee will be spurred to further explore and push it.

Four piezoelectric sensors are installed on the inner side of one of the Plexiglas layers with their plain side in direct contact with the Plexiglas surface. The location of each sensor is established so to induce a centroidal Voronoi tessellation on the Plexiglas surface [64]. This grants an optimal position of the piezoelectric sensors relative to the amount of sensors used and the data acquired. More specifically, each sensor lies on the center of mass of one of four tiles, thus minimizing the sum of distances between any point on the tile and the sensor.

The model of piezoelectric sensor we use was chosen heuristically after comparing and benchmarking a number of heterogeneous alternatives. Sensors were installed in the unit and exposed to three main strain categories: *touch*, *tap* and *thrust*. Among all piezoelectric elements tested, the Multicomp ABT-441-RC (Outer diameter: 27 mm, Resonant frequency: 4.2 kHz, leaded) and the Multicomp ABT-448-90-RC (Outer diameter: 35 mm, Resonant frequency: 2.9 kHz, leaded) proved to be the most appropriate for our purposes. In fact, using the language Processing (www.processing.org) to graph different parameters of each sensor under each condition, the Multicomp sensors, unlike their alternatives, showed categorically different responses depending on the type of strain. Both Multicomp elements, in fact, registered strain maxima that were higher for thrust than for tap, and higher for tap than for touch. This feature made them perfect candidates for filtering low strain manipulations by imposing a software threshold on strain maxima. All other things being equal given our requirements, we opted for employing the ABT-441-RC, its cost being about half of the ABT-448-90-RC.

### 4.2. Connections

**Figure 3.**
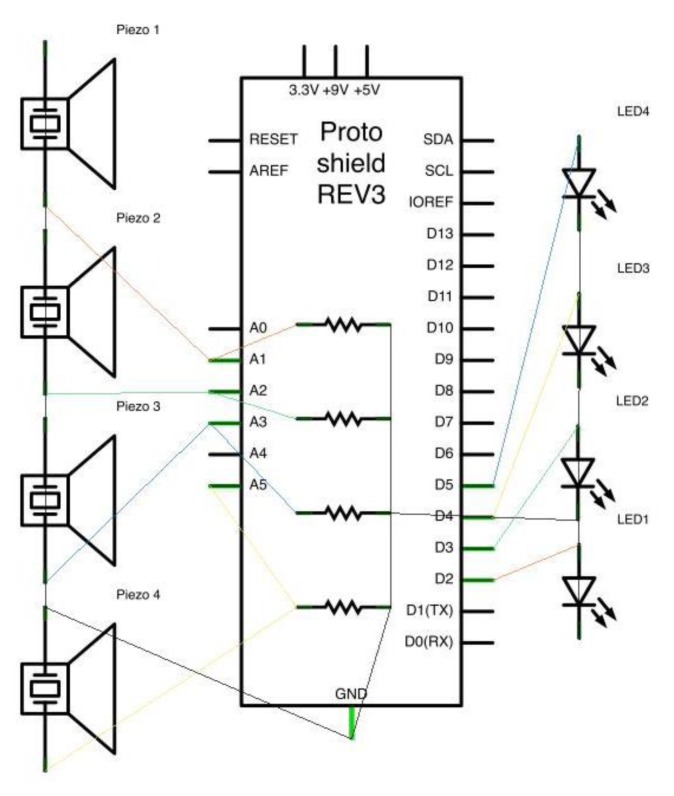
Schematics of the custom Arduino board built for the wired prototype.

Each piezoelectric sensor is connected through two unipolar cables (length: 1250 mm, outer diameter: 1.5 mm) to a custom-made board (see Figure 3 for schematics). Apart from the 8 inputs from the sensors (4 positives and 4 grounds), the board features resistors (resistance 1 MOhm), one for each piezo. Moreover, an LED (light-emitting diode) is assigned to each sensor, making it possible to check its operational state.

The custom board maps the positive pole of each piezoelectric sensor to an analog input slot on an Arduino board. All the ground signals collected on the custom board are mapped to a ground input on the Arduino. Similarly, each LED on the board connects to a different digital output on the Arduino at one end and a common ground at the other end.

### 4.3. Arduino: Features

This prototype makes use of an Arduino board for several purposes. First of all, a form of analog to digital conversion is required before sending data to a computer. The Arduino board accomplishes this by obtaining a reading of the four piezoelectric sensors. If readings are above a given threshold data are processed further, otherwise they are ignored. An additional Arduino script enables to light a LED indicating that readings above a given threshold were received from a particular piezoelectric element. This ensures the possibility of checking the correct functioning of all sensors. This is particularly important for the flawless functioning of the device, as a failure in one of the sensors could pass unnoticed and be masked by the readings of the remaining sensors. Finally, the Arduino board is in charge of data preprocessing. That is, part of the integer computations required by the device are distributed and performed by the Arduino.

The particular model of Arduino used in this project is an Arduino Uno, whose clock frequency is 16 Mhz. The baud rate, equal to 28,800, was established heuristically in order to find a good tradeoff between rate of serial transmission and possible delay due to overload. As the numerical values sent by the Arduino rarely exceed 2500, the chosen baud rate grants a lower bound on transmission rate of 1 sample per millisecond, which we deem adequate for our purposes.

### 4.4. Evaluation and Calibration of the Sensors

Our wired prototype sonifies strain provoked by sudden thrust movements. Choices on the combination of materials used (Plexiglas, foam and four piezoelectric elements) and the movements to be sensed by the piezos needed to be adapted to our particular animal sensing context. It was therefore necessary to calibrate the device. Every sensor's voltage is represented in the Arduino as an integer between 0 and 1023. Given that the Arduino adds up the readings received by the four sensors, the sum of all readings can theoretically be between 0 and 4092. As the simple gentle touching of the device produces readings up to 500, during calibration we imposed this number as the threshold below which Arduino would ignore readings.

The sensory module was put horizontally on the floor. A calibration weight of 5 kg was dropped on the module from different heights and the resulting data was recorded by a computer and further analyzed. The weight was dropped at heights of 20, 40, 60, 80, 100, 120 140, 160 and 180 cm, 5 times for each height, giving a total of 45 trials.

Every impact produced several actual readings plus those provoked by the rebound. The data was hence filtered to discriminate between trials and rebounds following a trial using a custom Python script. The script retained only readings which satisfied the following conditions on onsets and offsets. As trials were separated by at least 2 s their onset could be easily discriminated. Trial offsets were established by discarding all readings' sequences separated by more than 500 ms from the previous one. Hence, 63 out of a total of 855 readings were automatically dropped from the data set because they clearly corresponded to ricochet stress, being separated by more than 500 ms from the previous reading. The average length of each trial was 17.6 samples (*SD* = 4.87).

Our algorithms deal only with a few samples during each interaction, as waiting for a large number of samples would provoke a delay in playback. Hence we further analyzed only up to the first 10 samples per trial.

**Figure 4.**
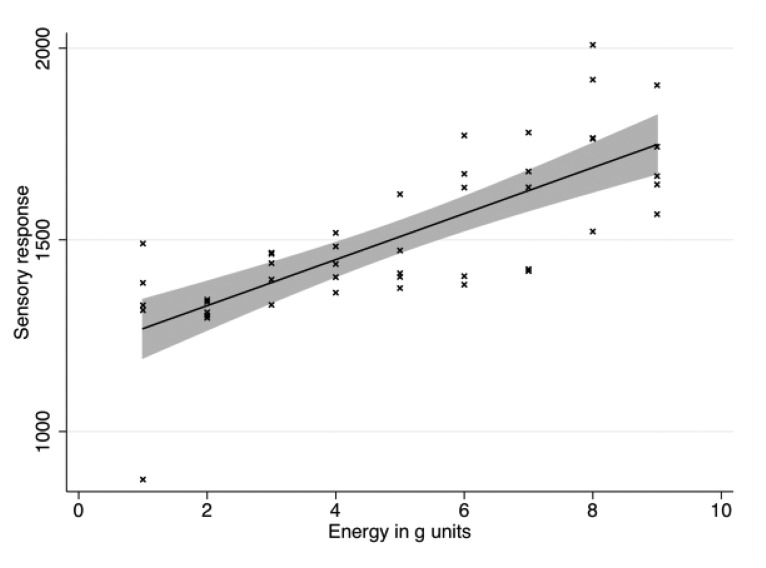
Each data point represents a sensor reading on the y-axis dependent on different levels of potential energy (just before the object starts falling) or, equivalently, kinetic energy (just before the impact) on the x-axis. Energy is plotted as multiples of acceleration of gravity g, and ranges between 1 g and 9 g. The line corresponds to the data's best fit using a linear regression model. The grey area is the 95% confidence interval.

The impact force of the weight on the module is approximately linear in the height of fall. Similarly, the current produced by one piezoelectric element is linear in the force of strain. Therefore, we hypothesized the sum of the readings from the four sensors to be approximately linear in the distance of fall. We therefore averaged within-trial readings and entered them in a linear regression model (statistical analysis was done in Stata 11.0). The model was significant [n = 45, F(1,43) = 57.35, p < 0.001] and could explain about half of the variance (Adjusted R-squared = 0.56). The fall height could significantly predict the sum of readings (t = 7.57, p < 0.001). The intercept was also statistically significant (t = 27.09, p < 0.001). The equation for the resulting strain is:

(1) r=1208.29+3.00*d+e

where r is the average reading, d is the fall distance in cm and e is the error. This result confirms our hypothesis about the existence of a linear relation between impact force/fall height and Arduino's readings (see Figure 4).

**Figure 5.**
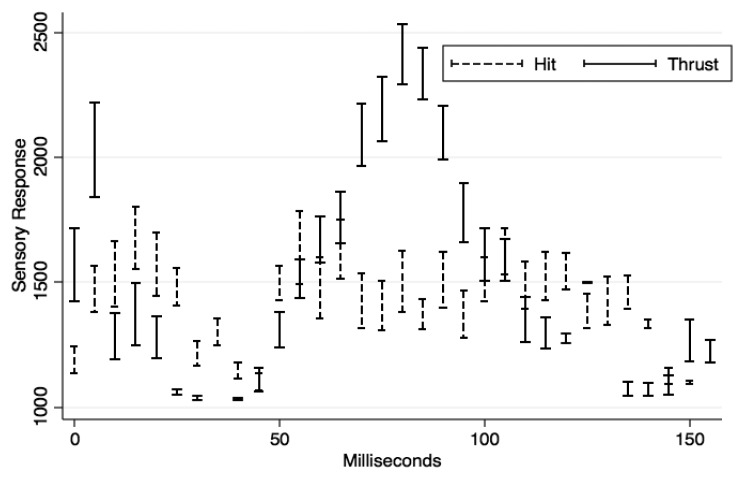
Mean and confidence interval of the time series of readings (y-axis) provoked by hit (superficial strikes, dashed lines) and thrust (strikes displacing the surface, unbroken lines) strain.

As real time playback is an important issue in our device, we decided to distribute computations between Python and Arduino. While Python is in charge of playback and logging, Arduino performs most of the threshold checks. To discriminate between *thrust* and *hit* types of strain, the Arduino implements a system of two thresholds separated by a delay. One of the main design challenges of this prototype was to make it selectively responsive to strain forces that would displace the outer Plexiglas inwards (*thrust*), as opposed to superficial strikes (*hit*). In order to systematically discriminate hits from thrusts using only the sum of the sensory readings, we systematically analyzed the times series of stress over time under the two conditions. The Arduino was set to send only samples above 1000 and a maximum of one sample every 5 ms. With a custom written Python script, we analyzed 59 repetitions of “hit” interactions and 59 repetitions of “thrust” interactions over time. For each type, the time series of each repetition and condition were aligned by onset and averaged over a time window of 5 ms (corresponding to the maximum time-resolution allowed by our Arduino settings). The resulting means and confidence intervals for both series are displayed in Figure 5.

Noticeable differences between series are (i) within the first 40 ms, when thrust is first associated with higher sensory readings, then with lower ones than hit, and (ii) between 70 and 95 ms, when readings associated with thrust are well above those recorded in the “hit” condition. In the first case, a local maximum for the “thrust” condition is reached at 5 ms. Moreover, at 5 ms the average reading for thrust is significantly higher than for hit (t-test for paired independent samples adjusted for unequal variances: *t* = −3.61, *p* < 0.01, Satterthwaite's degrees of freedom = 48.15).

To summarize, we found that a reliable way of distinguishing thrust from hit strain is to (a) further process all readings above a sensory response of 1000, (b) among these, discard all movements that have not exceeded a 1765 threshold after 5 ms (corresponding to the lower 95% confidence interval of the “Thrust” condition).

Taking this into account, we programmed the Arduino as follows. At every iteration, Arduino computes the sum of four concurrent readings and checks whether the current reading exceeds a given threshold (an “if” statement probing an inequality). If the current reading is large enough, Arduino waits 5 ms, reads again the four sensors and computes their sum. This second sum is then checked against a second threshold and is sent through the serial port only if it is larger than this threshold. After that, a refractory period of 300 ms before any further reading is imposed for two main reasons. First, it is advisable not to overload the serial port buffer. Second, stopping the sampling and transmission to Python is a straightforward way to avoid having one single movement elicit multiple sounds.

The computations performed in the Arduino can be seen as a form of filtering unimportant data and noise. This way, irrelevant data is not sent to the computer and processed further. Moreover, having Arduino perform these simple integer calculations constitutes a form of distributing tasks and having the computer available for further data handling and audio playback.

### 4.5. Python Software

The Arduino is connected to a computer through a USB interface. The computer runs a light Python script, composed of 7 functions spanning approximately 100 lines in total. The script is responsible for performing computations on the data, storing and sonifying them, that is, mapping them to particular sounds.

The Python script uses some internal and external modules. The datetime module is used to generate timestamps while the serial module is employed to communicate serially with the Arduino. The Pygame (www.pygame.org) external module is used to play sounds. First the script calls two Pygame *mixer* functions [mixer.init( ) and mixer.Sound( )] from the *Pygame* library to initialize the mixer and load the chosen sound. The script iterates through all the computer serial ports looking for an Arduino board. If an Arduino is found, it connects to it and starts listening with a 10 ms timeout. An important part of the script is the logging of parameters, sensory values and playback times. After establishing a serial connection, the script generates a comma separated values (csv) file and writes headers, so that all relevant sensory information can be recorded. Additionally, every time the thresholds (Table 2) and auditory parameters are modified and the script is restarted, a new header with the updated parameter information is written in a log file.

If contiguous sensor readings exceed critical thresholds (see previous section), the Arduino sends an integer consisting of the sum of all readings. The Python script receives and parses the serial input, ascertaining that it is a well-formed integer. This takes care of possible transmission errors, as non-integers are discarded. If a valid integer value is received, the script calls a *mixer* function [play( )] to play a pre-loaded sound. As soon as a sound is played, playback time [generated using a datetime( ) timestamp], together with volume and/or sensory reading are appended to a Python list. Once an experimental session is ended using a keystroke combination, the list is written to the csv log file.

The signal is sent from the computer soundboard to an external self-powered loudspeaker, so that the chimpanzee can hear the auditory feedback to its action. The loudspeaker is supposed to be placed outside the chimpanzees' enclosure, though directly behind the sensory module (to the extent possible, depending on regulations and conditions dictated by the animal facility).

### 4.6. Design Choices and Early Prototypes

Although the hardware and software solutions presented above may appear arbitrary, it should be noted that, in both cases, we opted for these solutions after testing different prototypes and comparing performances. The sensing module went through three different early prototypes, which featured diverse sensors contained in the same Plexiglas-foam structure presented here.

Wired prototype 1 simply featured a consumer-level piezoelectric element. This solution was quickly abandoned, as the sensory module could not systematically detect the type of stress we intended to measure.

Wired prototype 2 featured a 3-axes accelerometer. Accelerations corresponding to different stress types were compared and plotted over time. Even though thrusting and knocking elicited similar acceleration peaks in the data, their statistical properties over time markedly differed. In particular, the variance was distinctively higher for knocking stress, making this quantity a good candidate for discriminating between stress types. While developing the software, however, we found that discrimination on the basis of variance required a time window that was too wide for our solution to give auditory feedback in due time. The possibility of using an acceleration sensor was retained and piloted with a different method of detecting the nature of incoming stress.

With wired prototype 3 we piloted a hybrid between acceleration and strain sensors. Both a 3-axes accelerometer and a piezoelectric element were connected to the Arduino and their data sent to Python. As detailed above, the accelerometer was too sensitive for our purposes, sensing a number of vibrations provoked by simply knocking on the Plexiglas surface. Combining both readings would allow us to ignore acceleration data when strain data corresponding to knocking was present. Acceleration readings would otherwise be used to calculate the actual speed of displacement of the Plexiglas surface. Unfortunately, good data could be obtained with this method only at the cost of extensive computation, undermining one of the main requirements of the tool, namely speed.

## 5. Wireless Prototype

This prototype was designed to sonify another common behavior found in non-human primates, that is, manipulating and playing with mobile objects. In particular, several chimpanzees have been reported to actively manipulate the Kong toy (www.kongcompany.com) [55], which is the type of toy that we use here. Using this device, one can associate loud, unusual sounds to object shaking and throwing. This can also facilitate acoustic production for individuals that do not usually hit surfaces and walls (see Wired Prototype).

**Figure 6.**
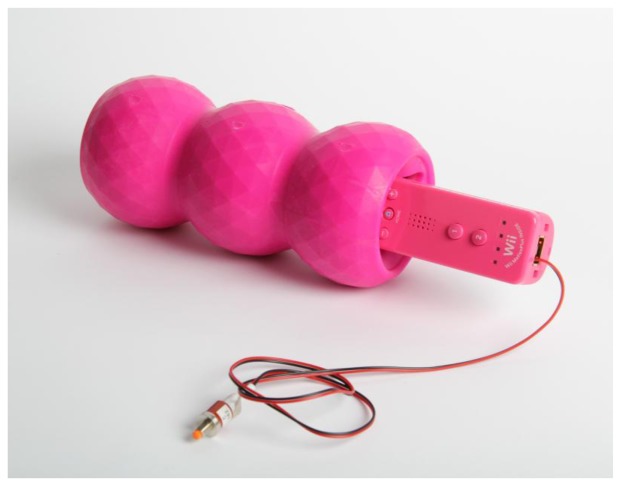
The wireless prototype. The cable, connecting our external custom synchronization button to the Wii Remote, can be hidden inside the Kong toy once the synchronization is accomplished.

The Wireless device is composed of a sensory module, namely a modified Wii Remote controller embedded in a Kong toy, and a computer with a Bluetooth interface (Figure 6). This prototype has several advantages. First of all, chimpanzees enjoy playing with objects and toys. Second, assembling this device requires less construction and electronic work than the wired prototype. All required components are available to buy online or in shops. Apart from a modification we operated on the Wii Remote in order to facilitate and speed up its interfacing with the computer, the only further work required for this prototype is software development in Max. Finally, depending on the foreseen application, a major advantage of this prototype resides in its wireless communication system. The sensory manipulable unit is in fact physically independent from, and connected via Bluetooth to, the computer.

### 5.1. Sensory Module: Kong

The Wii Remote is embedded in a gum toy (Kong Genius Mike), normally used as enrichment for dogs. The toy has approximately the shape of a cylinder (diameter: 82 mm, length: 180 mm) and is hollow, allowing the Wii Remote to be tightly fitted inside. Using Genius Mike as the outer part of our prototype has a number of advantages. First of all, its shape and physical characteristics make it an interesting object for non-human animals to play with. Chimpanzees have been reported to spend time playing and exploring Kong toys [55]. Second, the Kong is slightly deformable though resistant, spurring the animals' interest in manipulating it while ensuring protection for the Wii Remote located inside. Finally, the Kong is among the most well-known and widely used enrichments for dogs. This makes it an option easily available to buy and replace in case of damage.

### 5.2. Sensory Module: Wii Remote

The core of this wireless prototype consists in a mildly modified Wii Remote controller. We decided to base this prototype on a consumer-level product for financial and practical reasons. The Wii Remote combines a 3-axes accelerometer, a battery pack and a Bluetooth transmitter for an affordable price. After doing a preliminary feasibility study, exploring the possibility of building an equivalent unit out of basic components (Arduino board, analog accelerometer, wireless transmitter, *etc.*), employing the Wii Remote turned out to be a cheaper and less laborious solution than its alternative.

An additional advantage of using a Wii Remote is the amount of literature and programming libraries already available for this device [9]. The Wii Remote is one of the most common, consumer-level integrated sensory devices, and it has already been used in a number of projects dealing with augmented reality, robotics, music, cognitive psychology, *etc.* [7,8,10]. Considering its broad usage in a variety of scientific, medical and artistic projects, its advantages and disadvantages have long been pointed out and improvements were implemented in later versions [3,5,6,8,10,32,33].

Nintendo does not officially provide Wii Remote's technical specifications. However, given the interest generated by this device in different scientific communities, most specifications have been reverse-engineered. Here, we report some data provided by Lee [9]. The three-axis accelerometer is an ADXL330 (Analog Devices), granting a ±3 g sensitivity range. Data is updated every 10 ms at 8 bit per axis and transmitted through a Bluetooth connection using a Broadcom 2042 chip.

Our prototype features two minor modifications, which simplify battery charge and connectivity functions. The disposable batteries were replaced with NiMH 3A batteries, rechargeable through an ordinary USB port. This allows the controller's batteries to be recharged without extracting the Wii Remote from the Kong toy. Moreover, the internal connection for the button “SYNC” was disabled and substituted for a switch located outside the main body of the Wii Remote. As this button is normally used to synchronize the device with different software, placing it outside the main body grants an easy, painless setup every time the sensory unit is prepared for use.

### 5.3. Max/MSP Software

The software part for this prototype is programmed in Max 6. Max/MSP is a “visual programming environment”, where objects are connected, both visually and functionally, in order to form programs (patches and subpatches). Instead of typing lines of code, algorithms are constructed by combining elements in a graphical user interface, making Max an accessible option for people with little or no programming experience. Max is widely used among artists for audio and video live performance. As its architecture is optimized for real-time signal processing, Max is an ideal option for our project.

The Max patch we use here is composed of five subpatches, responsible for data acquisition, processing, parameter extraction, sonification and logging.

*Raw data acquisition* is handled by the patch disis.aka.wiiremote (http://ico.bukvic.net/) developed by Ivica Ico Bukvic, based on aka.wiiremote (http://www.iamas.ac.jp/~aka/max/) by Masayuki Akamatsu. This patch enables Max to connect to the controller and to acquire the raw acceleration data in three dimensions.

The second subpatch is responsible for *data processing*. First of all, for each axis, the patch computes the running difference between consecutive accelerations over time (delta). These values are then used to calculate the Euclidean norm. These two computations are equivalent to calculating the change in acceleration over time (*jerk*) in the three-dimensional space. We focused on change in acceleration, rather than on absolute acceleration values, for practical matters. As this prototype is wireless, the chimpanzee will realistically walk while carrying it. Thus, periods of walking could be interspersed with bouts of object shaking, and our device needs to be able to discriminate between them. By only taking into account jerk, the fairly constant speed of walking will be ignored, while more abrupt variations in acceleration will be processed further. This is intended as a first filter on the data, towards the extraction of shaking and jerking movements. It is worth noticing that, as only jerk is used, there is no need to take into account the acceleration of gravity when processing data further [3,8].

**Figure 7.**
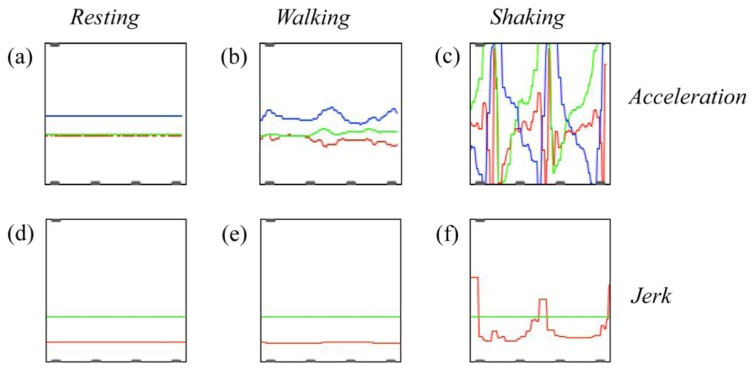
Acceleration readings (top row) from the wireless prototype and computed jerk values (bottom row) under three different conditions (columns).

Our third subpatch deals with *parameter extraction*. Its input is the computed jerk value, received every 10 ms while its output is a Boolean (specifically, a “bang” in Max syntax). This subpatch tests its input against two thresholds. A first “activation” threshold ensures that the jerk is above a critical value, in which case a “bang” is forwarded. Both the activation threshold and the incoming jerk value can be graphically displayed in real time (using a subpatch adapted from disis.aka.wiiremote). This enables visual inspection of the incoming signal and adjustment of the threshold according to the current magnitude of the parameter (see Figure 7). Setting a threshold slightly lower than the peak jerk value associated with the movement of interest can make the patch partially predictive [3,8].

Once activated, the bang reaches a timer and, after a comparison with the current time value, the timer is restarted. Verifying whether enough time has elapsed from last activation outputs a Boolean, which is sent to the sonification patch. The timer's characteristics ensure two desirable properties of our device: (i) no sound can be played within a delta time from the previous sound and (ii) if several activations are received in a short time interval, only the first one is sonified, with the timer being continuously reset. Property (i) prevents the device from producing two sounds when only one movement occurred while (ii) ensures that the production of different sound units is separated by periods of no or little movement.

The *sonification* part is kept to a minimum and is in charge of loading and playing chosen sounds. It is, however, possible to modify and expand it. Features computed in the data processing patch (such as acceleration magnitude and direction) could be sent to the sonification patch, so as to map particular movements to specific sonic features. In the simplest case, the magnitude of the acceleration could be mapped to the volume of the sound being played. An additional movement counter could for instance be added, so as to map movements within a cluster to different sounds depending on their order of occurrence. Moreover, Max/MSP is designed to enable a fast implementation of acoustic effects, such as echo, delay, *etc.*, which we foresee as a particularly useful feature in our future experiments.

The last patch is responsible for *logging the data*. It receives the parameter extraction patch's output and writes it to a text file. It associates a time stamp, with 10 ms precision, to every input value and immediately writes both pieces of information as a new line to a .txt or .csv file. Additional data, such as the name of the sound played or acoustic parameters can be automatically added to the lines of text.

In the current version of the prototype, the sound is broadcasted through an external self-powered loudspeaker. This should be located outside the animals' enclosure and connected to the computer through cables. For small enough chimpanzee enclosures, this solution can be adequate. Even though the Wii Remote contains a loudspeaker, its low sound quality makes it unsuitable to play rich, structured sounds. We are currently working on a solution to send the audio signal back to the sensory module via radio (to avoid overloading the Bluetooth transmission) and to play sounds using an additional loudspeaker that would be contained in the Kong toy.

### 5.4. Testing the Prototype: Preliminary Results

We tested the operational range of the sensory unit indoors. No decrease in, or loss of, signal was reported, even at a distance greater than 20 m with several walls interposed. Using the prototype in an environment where the frequency band is shared with other devices could result in a decrease in the transmission range. However, this should not be a concern in animal facilities, where most radio devices—walkie talkies used by animal keepers and personnel—transmit at lower frequencies than Bluetooth ones. Unlike the wired prototype, which consisted of a new combination of technologies, the wireless prototype was not specifically calibrated. Given that the parameter used to produce sounds is based on the change in acceleration over time (see Section 5.3), any discordance between sensory readings and real accelerations, as long as it is a linear, systematic error, will not affect the computed jerk values and therefore will have no effect on sound production [65]. Hence, calibration was deemed unnecessary for our purposes.

As a preliminary feasibility test, we compared the readings obtained at rest to those obtained while walking with the device or when shaking it (Figure 7). In graphs a-c, accelerations along the 3 axes are plotted over time. The red line depicts accelerations along the x-axis (Wii Remote's coordinate system), the green line corresponds to the y-axis and the blue line refers to the z-axis. (Referring to the position of the Wii Remote in Figure 6, these axes correspond to upwards-downwards accelerations for x, leftwards-rightwards for y and towards/away from the observer for z.) In graphs d-f, jerk values obtained over time are depicted (red line), together with a sound activation threshold (green line).

The first column shows readings while the sensory module is at rest. Minimal variations in acceleration (top, Figure 7a) are not present in the corresponding jerk plot (bottom, Figure 7d).

The second column depicts 3-dimensional accelerations (Figure 7b) and jerk (Figure 7e) when walking with the device (human being). Accelerations are present (top) and possibly biased, the most obvious example being the blue line shifted vertically due to the acceleration of gravity. The jerk (bottom), however, is minimal and well below the depicted threshold.

The third column shows accelerations (Figure 7c) and jerk (Figure 7f) while shaking the device three times in succession (human being): here three potential activation events occurred. In order to be sonified, however, each potential event needs to pass the time activation threshold (see Section 5.3).

This preliminary test suggests that jerk can indeed be used to discriminate between movements associated with walking and movements associated with shaking the device. Indeed, an appropriate sound activation threshold can be selected such that walking does not trigger sound production but shaking the device leads to sound production. Chimpanzees show individual differences relative to gait, and their locomotion alternates between bipedal and quadrupedal [66]. Hence, the particular value of the sound activation threshold will have to be adjusted to the specific individual taking the experiment.

## 6. Pilot Work with Chimpanzees

A crucial step for validating our prototypes was to test them with non-human primates. Here we describe pilot work done with a group of chimpanzees at Edinburgh Zoo, Scotland, UK. We decided to focus on piloting the wired prototype only. This is because the vastness of the chimpanzee outdoor enclosure and the species-adequate structure of the indoor enclosure pods did not allow us to follow a focal chimpanzee transporting the wireless device. This entailed the risk that both, visual and radio contact with the device might have been lost repeatedly.

### 6.1. Methods

#### 6.1.1. Study Species and Study Site

Budongo Trail, located at Edinburgh Zoo, houses 18 chimpanzees (10 females and 8 males) socially in an 1,832 m^2^ outdoor enclosure and three interconnected 12 × 12 × 14 m indoor enclosures [67]. The animals have *ad libitum* access to water and are fed four times a day. Research with the chimpanzees is strictly non-invasive. The animals take part in experiments voluntarily and can leave them at any moment. Before taking place, this pilot study was approved by the scientific board of Budongo Trail, and did not entail any ethical concerns. The nature of the study was observational and no invasive methods were applied.

#### 6.1.2. Device Setup

**Figure 8.**
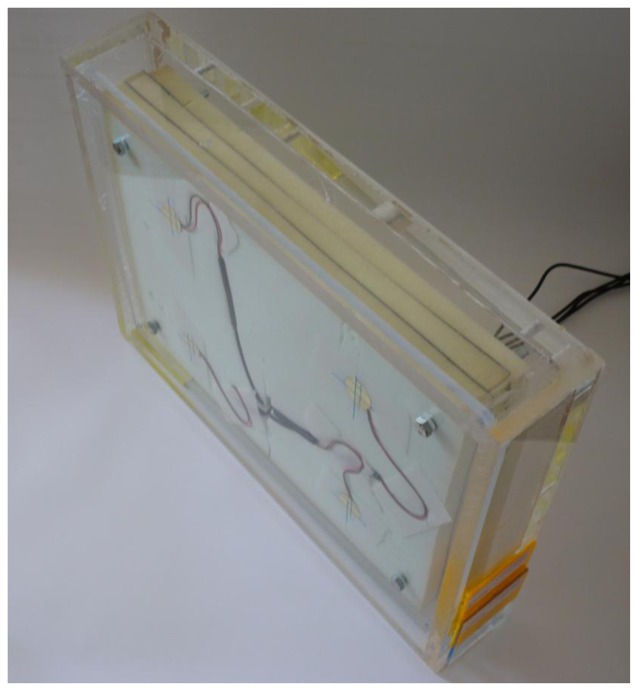
Wired prototype, after slight modifications to fit the pilot testing location. A thicker inner Plexiglas layer granted better resistance once screwed to the chimpanzees' enclosure. Moreover, as the device had to be placed in the enclosure in its entirety, “protection walls” were built to avoid chimpanzees' contact with the solid foam.

The wired prototype was piloted with the Budongo chimpanzees. We slightly modified the prototype to adapt it to the specific testing location (See Figure 8). The inner Plexiglas layer, containing no sensors, was replaced by a thicker (15 mm) concave one (to protect the sides of the device). The outer layer's dimensions (width and length) were increased by 36 mm each, while the Plexiglas' thickness was left unchanged. Plugs were added to the connection cables so they could be passed through multiple enclosure elements. Sounds were broadcasted using an active loudspeaker (JBL Control 2P) placed approximately 1 m behind the device.

During the pilot session described below, in order to maximize the likelihood of interaction with the device, the Arduino thresholds were lowered, so that even a delicate touch would elicit a sound. The device was installed and fixed on a wire mesh in one of the core chimpanzees' whereabouts, which granted that all individuals could have access to the device. Chimpanzees could access this area from two sides on two different floors.

### 6.2. Pilot Session and Data Collection

After installing the device in the animals' enclosure, chimpanzees were allowed to enter and interact with the prototype. Given the exploratory nature of this pilot experiment, we decided to keep the sound intensity and type mostly constant (snare drum). However, during brief periods of time, the sound mapping was altered, in order to explore and probe future research directions. Throughout the approximately 33 min long session, three different types of sounds were associated to chimpanzees' manipulations: snare drum (69.5% of the time), Hi-hat drum (1.5%), and human spoken syllables (17.5%). The remaining 11.5% of the time, either no sound was associated to manipulations or no chimpanzee interacted with the device. The pilot session was recorded on video from which behavioral and interaction patterns were coded [68,69] using ELAN [70–72]. The following behaviors towards the device were coded for:

- approaches to and recessions from the device (within a 2 m distance),
- smelling, licking, looking, gently touching, grabbing and climbing,
- using additional tools (such as sticks) to interact,
- poking with finger or hand,
- pushing with shoulder, head, hands, feet or other body parts,
- hitting with hand or arm,
- pant-hoot display, including kicking or punching.

A custom Python script was used to extract the data from ELAN [70–72] annotations and fill in Table 3.

### 6.3. Results

A total of 12 individuals (reported as C1 to C12 in Table 3) voluntarily approached the device and interacted with it. Each of these chimpanzees spent between 6 s and 9 min in proximity of the device. The overall time spent by the group in proximity of the prototype exceeded 38 min: at several points in time, two (or more) individuals were in proximity of the device. All 12 individuals who approached the device also showed exploratory behavior.

At a group level of analysis, the behavior most frequently recorded was pushing (Table 3). Five individuals pushed the prototype eliciting sounds. Two individuals (C4 with 75 and C11 with 90 pushes) in particular produced sounds by pushing. The second most observed behavior was poking. All 12 chimpanzees who interacted with the device showed this behavior. Two individuals hit the device, one of them 51 times. Five out of the 12 chimpanzees interacting with the device used a tool (such as a stick) to poke on the surface or between gaps of the prototype. Two chimpanzees used the device for dominance displays, each of them twice.

The coded behaviors can be divided into three main behavioral categories: (i) explorative behaviors (poking, tool use) [73,74], (ii) play behaviors (pushing and hitting) [75,76], and (iii) dominance behaviors (displays with hits or kicks) [75]. Applying this categorization, the behavioral observations indicate that the chimpanzees used the device foremost for play, followed by explorative behavior, and then dominance behaviors.

### 6.4. Discussion

Overall, the pilot session showed that the wired prototype fulfills its intended purpose. The prototype withstood vigorous manipulation, including kicks and punches. While exploring the device, chimpanzees stood on it without any damage to the unit. This suggests that both the prototype itself and our solution for fixing it to animal enclosures are stable and resistant.

Interaction rates with the device were high both in the number of participating individuals, and in the total amount of interactions observed. The majority of the chimpanzees (12 out of 18 individuals) showed some sort of interaction with the device within only 33 min of access. This suggests that the wired prototype represents an object of interest for our study species. This is a crucial prerequisite for the conduction of further experiments on acoustic pattern production in chimpanzees.

One of the most common ways of interacting with the prototype was to poke the outer Plexiglas layer. More specifically, all chimpanzees touched the sound-producing surface of the device with their hands or fingers. This can be interpreted as explorative behavior related to getting acquainted to the novel object. Further *explorative behavior*, such as looking at, licking, or touching the device, was observed at high rates. Several individuals also used tools to further interact with the device. In the long term however, as the chimpanzees familiarize themselves with the device, we predict a decrease in explorative behaviors. Hence, we argue that no experimental design should relate to these kinds of manipulations of the device.

Pushing the outer layer was the most frequent interaction method recorded overall, though only used by six chimpanzees. Hitting the prototype was a relatively infrequent behavior, found only in two individuals. This can be partly due to the novelty effect of the device. As explorative behaviors are expected to decrease over time, other behaviors such as hitting and pushing are predicted to increase. Pushing and hitting behaviors can be interpreted as *play behavior*. This interpretation is supported by the fact that the chimpanzees interacting with the prototype often showed species-specific play face (open mouth and teeth shown) [75]. For instance, individual C4 climbed on the device, held himself onto the wire mesh and started a series of pushes with his feet. The pushing was accompanied by a facial expression typically associated to play behavior in chimpanzees. Play face was also observed when individual C8 sat in front of the device, hitting it multiple times in a row.

Play behavior can be a good starting point to build upon when designing experiments, since: (1) the amount of playful interactions with the device can be expected to increase over time, (2) using the device in a playful manner suggests that a positive self-rewarding mechanism not only increases the total amount of interactions, but also enlarges the behavioral repertoire (*i.e.*, how the device is used). Experimentally manipulating sounds that are played back to the study subjects can further enhance the chimpanzees' interest and positively feed back to the exhibition of play behavior. In this respect we see also a positive potential welfare aspect for captive chimpanzees. Using the device as an enrichment tool might eventually increase the animals' wellbeing in captive environments.

Two individuals used the device to *display*, performing a series of loud vocalizations (pant-hoots), which culminated in hitting the device with upper or lower limbs. Only male chimpanzees showed this type of behavior. Considering the relatively short access time (33 min), this frequency of displays can be considered high. Allowing chimpanzees open access to such a device would likely result in its usage for such dominance displays. Hence we argue that this behavioral category offers another area of interest for future experiments.

## 7. Sonification: Possible Criteria and Future Directions

The device was used by a large proportion of chimpanzees and in a variety of ways. This suggests that its shape and physical characteristics will be an asset in engaging chimpanzee participants to take part to future experiments. In light of the pilot session described above and the available literature on chimpanzees' behavior, we can now put forward possibilities of movement-sound mappings, which can be implemented in our prototypes in the near future.

The sound associated to manipulations was mostly that of a snare drum at constant volume. While one of the individuals was producing the snare sound, we artificially changed the mapping to a (less prominent) hi-hat sound. This elicited an unusual vocalization, comparable to an expression of disappointment. Mapping to human-spoken syllables, instead, did not appear to elicit particular reactions. Even though these only constitute anecdotic instances, we believe that manipulation of sound mappings could have a lot of potential. Future experiments, controlling for and counterbalancing the type and proportion of sounds associated with manipulation, will be able to inform us about chimpanzees' preferences for movement-sound and emotional state-sound mappings.

During the pilot, interactions of different strength were shown. Displays, from the point of view of strain, were harder than play or explorative behavior. These two types of (soft and hard) manipulations should however be regarded as extremes of a continuum, rather than clear-cut categories. Taking into account chimpanzees' biology, displays are usually associated with increased levels of arousal. This consideration should be included when designing sonification paradigms.

Both the type of interaction and the context in which it is performed should inform the movement-sound mapping. The wired prototype can discriminate *hit* from *trust* movements. The former manipulations are often associated with exploratory and play behavior while the latter with pant-hoot displays. Hence, a first possibility could be to play the same sound in the two conditions, while assigning a higher playback intensity level to the thrust condition. Alternatively, or in addition to the intensity manipulation, different sounds could be assigned to the various conditions. The spectral properties of the sounds should reflect this distinction: sounds rich in low frequencies, with short attack time, rough and possibly noisy (mimicking spectral characteristics of natural chimpanzee drumming during pant-hoot displays) could be assigned to the thrust condition. In order to generate a contrast, sounds with opposite characteristic could be assigned to exploratory manipulations.

Other possibilities could be to only manipulate spectral characteristics when chimpanzees play with the device, and sound intensity while chimpanzees perform dominance displays. While sound novelty and variety may be more appropriate when chimpanzees are in a playful mood, unexpectedly high or low intensity could elicit interesting reactions during dominance displays.

As mentioned above, we did not have the opportunity to pilot the wireless prototype with chimpanzees. However, we could observe chimpanzees playing with the gum toy used as outer shell for the prototype. Extrapolating from the pilot session, a continuum of sensed states could be mapped to sound. Assuming that the chimpanzees' strength in manipulating the device will increase concurrently with their arousal levels, the sound should vary accordingly. Mapping higher *jerk* values to increased sound intensity and sound roughness would be the first straightforward possibility.

All these options would have the advantage of: (i) starting a positive feedback loop between chimpanzees' arousal and sound intensity, and (ii) not scaring away explorative individuals with incommensurately loud or potent sounds. Once this kind of mapping is in place, however, we could systematically invert the mapping, so to violate the animals' expectations and record the resulting behavior.

We recognize that this is a modest range of possibilities in comparison to human sonification projects. However, this is also the first attested attempt to connect machine interaction, auditory cognition and sonification in a non-human primate.

## 8. Conclusions

Over the last decade, music performance and cognitive experiments have been increasingly relying on the use of human-computer interaction technologies [7,27,30]. Though many devices have been specifically produced and optimized for human use, the field of animal cognition still lacks the development of enough ad-hoc technological tools [35,49,50]. In particular, although many possibilities exist for sonifying human movements, none has been developed in the field of non-human primate research.

Here we present two prototypes that we developed for mapping chimpanzees' object manipulation to sounds, using acceleration and strain sensors. Our prototypes will serve to test crucial hypotheses on primate auditory cognition, musicality and evolutionary development [22,23,25,77]. New devices had to be created, as tools currently available for human research lack a number of features necessary when working with chimpanzees. In particular, the sensory units of our prototypes are resistant to robust handling, modular, low-voltage and relatively cheap [56]. These qualities, foreseeing a vigorous manipulation by chimpanzees, avoid the destruction of the entire device, prevent electrocution, and allow for easy replacement of parts.

The software part of our prototypes has a number of interesting qualities, which make it particularly suitable for auditory experiments. First of all, the simplicity of our algorithms grants a short delay between sensing and auditory feedback. Second, acoustic parameters can be quickly adapted to maximize the primates' interest in the device. Finally, all relevant sensory data can be logged in a computer to enable more efficient data analysis.

Our two prototypes satisfy a number of desiderata though differ in several respects. Our wired prototype is supposed to be mounted on a vertical surface and employs cables to connect to a Mac computer via an Arduino board. It is designed to acoustically respond to thrust and has a ricochet property, which encourages such movement. Four piezoelectric elements inside the prototype are responsible for strain data acquisition. This prototype has the advantage of being particularly cheap to develop, though it requires some electronic and construction work.

Our wireless prototype consists in a modified Wii Remote embedded in a gum toy and connected to a Mac computer via Bluetooth. The data from the acceleration sensor in the Wii Remote is sonified in case of shaking and jerking movements of a given magnitude. This prototype has the advantage of being wireless and requiring little development work, as its components can be easily bought and assembled.

These prototypes are a first step towards testing a range of hypotheses on the human specificity of rhythmic abilities among primates. In light of the pilot study presented here, we intend to further adjust our designs to chimpanzees' and experimental needs. Successively we intend to test, among others: preference for movement-sound mappings, multimodal entrainment to a steady pulse, discrimination and preference for different metric and grouping structures, and rhythmic imitation capabilities.

The fact that the wired prototype was used for dominance displays suggests that the prototypes may enable researchers to tap into chimpanzees' auditory cognition and spontaneous social behavior. Jane Goodall reports the story of a chimpanzee in the wild who, through the fortuitous discovery of a resonating barrel, climbed up the dominance hierarchy by being the loudest drummer in the party [78]. Goodall's report hence hints at the importance of sonic interactions in chimpanzees' social life. Together with our pilot data, it also suggests that future experiments on non-vocal sound production in chimpanzees can be designed without losing ecological validity.

The devices presented here are noteworthy for several reasons. To our knowledge, they constitute the first attempt at building a sensory device specifically designed for chimpanzee-computer cognitive interaction. Their development was markedly interdisciplinary, benefitting from knowledge both in human-computer interaction and animal cognition. Finally, these devices will serve for several crucial experiments dealing with human-chimpanzee comparative cognition. These experiments will allow to substitute conjectures and key hypotheses on the purported uniqueness of human musicality and language abilities with solid empirical data [25,56,77].

## Figures and Tables

**Table 1.** The first column shows critical requirements and whether these are satisfied by objects/surfaces naturally present in the animals' enclosure (second column), commercially available electronic drum sets (third column) and our prototypes (last column).

*Requirements*	**Object/wall in enclosure**	**Commercial electronic drums**	**Our prototypes**
Sound production when manipulated	✓	✓	✓
Precise data recording	✗	✓	✓
Physical resistance	✓	✗	✓
Weather resistance	✓	✗	✓
Low-voltage	✓	✓	✓
Modular	✗	✗	✓
Spurring interaction	✓	✗	✓

**Table 2.** Parameters of the prototypes that can be adjusted by the user. Both prototypes have a customizable time threshold, *i.e.*, a period during which sensor readings cease. The wired prototype has an adjustable strain threshold for its activation and a second threshold, above which a sound is triggered. The wireless prototype features an adjustable threshold on the variation in acceleration, above which a sound is played.

	**Time threshold:**	**Activation threshold on:**	**Sound production threshold on:**
**Wired**	Present	Sum of readings	Sum of readings
**Wireless**	Present	Difference in acceleration	Coincides with the “Activation”

**Table 3.** Duration of proximity to the wired device and frequency of interaction (by interaction type) performed by 12 individual chimpanzees.

**Name**	**Proximity (s)**	**Poking**	**Tool**	**Push**	**Hit**	**Display**
C1	211	12	2	0	0	0
C2	140	3	0	0	0	0
C3	122	19	0	4	3	0
C4	211	32	3	75	0	0
C5	32	1	0	0	0	0
C6	68	2	0	0	0	2
C7	517	1	0	0	0	0
C8	288	31	5	35	51	2
C9	458	20	10	10	0	0
C10	6	1	0	0	0	0
C11	243	25	1	90	0	0
C12	20	2	0	3	0	0
*Total*	2,316	149	21	217	54	4
